# Impaired selective renal filtration captured by eGFR_cysC_/eGFR_crea_ ratio is associated with mortality in a population based cohort of older women

**DOI:** 10.1038/s41598-022-05320-w

**Published:** 2022-01-24

**Authors:** Linnea Malmgren, Fiona E. McGuigan, Anders Christensson, Kristina E. Akesson

**Affiliations:** 1 Department of Clinical Sciences, Lund University, Malmö, Sweden; 2 Department of Geriatrics, Skåne University Hospital, Malmö, Sweden; 3 Department of Orthopaedics, Skåne University Hospital, Malmö, Sweden; 4 Department of Nephrology, Skåne University Hospital, Malmö, Sweden

**Keywords:** Kidney diseases, Biomarkers, Translational research

## Abstract

Deranged renal filtration of mid-sized (5–30 kDa) compared to smaller molecules (< 0.9 kDa) results in increased plasma levels of cystatin C (cysC) compared to creatinine resulting in a low eGFR_cysC_/eGFR_crea_ ratio. A ratio below 0.6 or 0.7, is termed shrunken pore syndrome (SPS), which in patient based studies is associated with mortality. Reference values for eGFR_cysC_/eGFR_crea_ ratio, the prevalence of SPS and the consequence of low eGFR_cysC_/eGFR_crea_ ratio in the general, elderly population are unknown. 75-yr old women (n = 849) from the population-based OPRA cohort, followed for 10-years had eGFR calculated with CKD-EPI study equation, and eGFR_cysC_/eGFR_crea_ ratio calculated. Mortality risk (HR [95% CI]) was estimated. Women with sarcopenia or on glucocorticoids were excluded. Almost 1 in 10 women (9%) had eGFR_cysC_/eGFR_crea_ ratio < 0.6 at age 75 and this did not increase appreciably with age. Women with ratio < 0.6 had higher 10-yr mortality risk compared with ratios > 0.9 (HR_adj_ 1.6 [95% CI 1.1–2.5]). In elderly women eGFR_cysC_/eGFR_crea_ ratio < 0.6 is common and associated with increased mortality. Our results confirm patient-based findings, suggesting that identifying individuals with SPS may be clinically relevant to assessing mortality risk in the elderly.

## Introduction

Decreased kidney function results in lower clearance and increased plasma concentration of glomerular filtration rate (GFR) markers, for example cystatin C (cysC) or the commonly used creatinine. In 2015 Grubb et al. described a new syndrome affecting kidney filtration, characterized by a disturbance in the ability to filter larger i.e. 5–30 kDa sized molecules. Termed Shrunken Pore Syndrome (SPS), this results in elevated plasma concentration of middle sized proteins such as (cysC (13.3 kDa) compared to smaller molecules < 0.9 kDa, such as creatinine (0.113 kDa) or water (0.018 kDa)^1–3^, resulting in a low eGFR_cysC_/eGFR_crea_ ratio.

**Figure 1:**
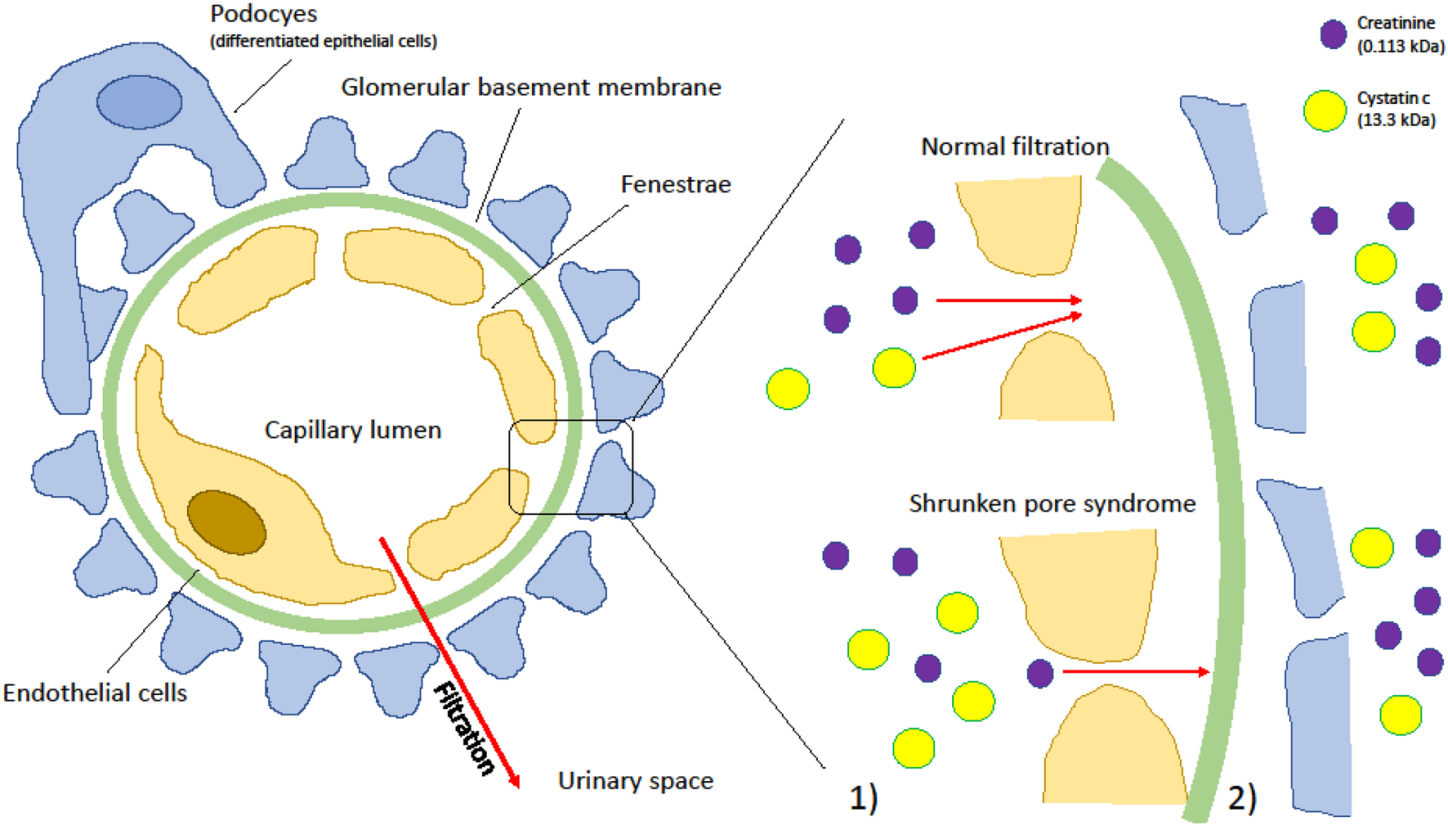
Two possible mechanisms of SPS: (1) decreasing of the pore size and (2) thickening of the glomerular basement membrane.

The kidney is a complex organ and no generally accepted 3D-model of the glomerular filtration barrier exists (4). However, two pathophysiological models are currently suggested to explain the affected filtration quality; decreased pore size and, or thickening of the glomerular basement membrane (GBM) (Fig. 1). A thickening of the GBM would lead to an increased diffusion length of cysC, thus affecting the plasma concentration^1,5^. While the syndrome has been associated with mortality, both in individuals with normal kidney function and those with reduced GFR^4,6–8^, to date only one study has follow-up above five years^6^, hence, the association to long-term mortality is unknown.

Given the limited literature to date^3^ there are many questions to be answered. The first of these surrounds diagnosis. Plasma concentrations of creatinine and cysC, and therefore their respective estimations of GFR (eGFR), differ in SPS. Since creatinine levels are affected by age and sex, to enable comparisons between individuals, diagnosis is therefore based on the ratio of eGFR_cysC_ to eGFR_crea_, as the eGFR study equations take these two factors into account. However, SPS should only be diagnosed in the absence of non-renal factors influencing creatinine and cystatin C e.g. sarcopenia and glucocorticoid treatment^1,3^. As of yet, no absolute cutoff in the ratio of eGFR_cysC_/eGFR_crea_ has been defined^2^. While most studies use a ratio of < 0.60 or 0.70, some suggest that specific clinical settings and conditions could warrant alternative cutoffs^4,6,9,10^. For example, age, eGFR study equation and comorbidities are all factors that could affect the eGFR_cysC_/eGFR_crea_ ratio.

The second of these questions is prevalence. Reported prevalence of SPS varies from 0.2 to 36%, but these estimates are primarily based on studies in selected patient cohorts, predominantly with heart disease^2,7,9,11–13^. The ratio of eGFR_cysC_/eGFR_crea_ and hence prevalence in the general population is as yet unknown. Although one paper investigated healthy seniors, it is unclear whether the results are generalizable to a wider elderly population (based on the recruitment strategy and additionally because, while underweight participants were excluded this is only an indirect measurement of sarcopenia)^4^.

The next of these questions is chronicity. This disruption in glomerular sieving is closely linked to cardiovascular outcomes^12,13^ but it has also been identified in pregnant women and even children^14,15^, suggesting that altered ratios of eGFR_cysC_/eGFR_crea_ may not just affect the elderly or those with cardiovascular comorbidities. It also raises questions about the stability of the deranged filtration. Although the data from pregnant women indicate a disturbed eGFR_cysC_/eGFR_crea_ ratio may be reversible, this has not been confirmed at other ages or in other settings^3^.

The present study aimed to address some of these substantial gaps in knowledge through a longitudinal study, investigating 1044 75 year old women who attended three consecutive follow-ups over 10-years and for whom information on sarcopenia and glucocorticoid treatment was available. Our aims were (1) to longitudinally investigate eGFR_cysC_/eGFR_crea_ ratio in community dwelling women, suggest reference values for healthy older adults and determine prevalence of SPS and (2) investigate the association between eGFR_cysC_/eGFR_crea_ ratio using different cut offs previously described in the literature and mortality over ten years in a population based setting.

## Results

The mean eGFR_cysC_/eGFR_crea ratio_ (CKD-EPI) of the 849 women included in the analyses at study start (age 75) was 0.86 (ranging 0.33 to 2.22) based on the individual markers eGFR_cysC_ (64.8 mL/min/1.73 m^2^) and eGFR_crea_ (75.3 mL/min/1.73 m^2^). High blood pressure was common, with 40% on treatment, while 6% reported diabetes, 18% CVD and 14% were current smokers (Table 1).

**Table 1:** Baseline characteristics of the OPRA cohort of women all aged 75 at inclusion (n = 849).

Characteristics	Mean	(SD)
Age (years)	75.2	(0.14)
Body Mass Index (kg/m^2^)	26.3	(4.0)
Weight (kg)	67.9	(10.9)
Height (cm)	161	(6)
p-cysC (mg/L)	1.1	(0.3)
p-crea (µmol/L)	68.7	(13.5)
eGFR_cysC_ (mL/min/1.73 m^2^)	64.8	(17.1)
eGFR_crea_ (mL/min/1.73 m^2^)	75.3	(12.5)
eGFR_cysC_/eGFR_crea_ ratio	0.86	(0.19)
p-CRP* (mg/L)	1.8	(2.7)
p-calcium (mmol/L)	2.4	(0.1)
p-albumin (g/L)	40.9	(2.4)
p-phosphate (mmol/L)	1.1	(0.2)
s-PTH* (pmol/L)	4.2	(2.2)
s-25(OH)D3 (nmol/L)	62.5	(19.2)
p-TSH* (g/L)	1.7	1.6
p-homocysteine* (µmol/L)	13.9	5.4
s-folate (nmol/L)	22.1	10.8
p-cobalamine* (nmol/L)	308	171

	n	(%)
Treatment for high blood pressure	321	(40)
Cardiovascular disease**	151	(18)
Diabetes	53	(6)
Current smoker	116	(14)

*Median with interquartile range.

**Cardiovascular disease defined as hypertensive treatment in combination with an anticoagulant or lipid-modifying agent, or treatment with only vasodilators.

Prevalence of SPS is dependent on the selected threshold of the eGFR_cysC_/eGFR_crea_ ratio. Cross sectionally at age 75 (n = 849), a total of 80 women (9%) had an eGFR_cysC_/eGFR_crea_ ratio < 0.6, while 165 (19%) had an eGFR_cysC_/eGFR_crea_ ratio < 0.7. Five years later at age 80 (n = 569), 25 women (4%) and 134 (24%) had a ratio < 0.6 and < 0.7, respectively. At the last follow-up investigation (age 85, n = 286), 24 women (8%) had an eGFR_cysC_/eGFR_crea_ ratio < 0.6, while 70 women (24%) had a ratio < 0.7 (Table 2). Consequently, by the most common definition (an eGFR_cysC_/eGFR_crea_ ratio < 0.6), prevalence of SPS in elderly women amounts to one in ten at age 75 using the CKD-EPI study equation.

**Table 2:** eGFR_cysC_/eGFR_crea_ ratio and proportion with shrunken pore syndrome* at ages 75, 80 and 85.

Age	eGFR_cysC_/eGFR_crea_ ratio		
	< 0.6	0.6–0.69	< 0.7
75	80 (9%)	85 (10%)	165 (19%)
80	25 (4%)	109 (19%)	134 (24%)
85	24 (8%)	46 (16%)	70 (24%)

Age 75; n = 849, age 80 n = 569, age 85; n = 286.

*Defined using the Chronic Kidney Disease Epidemiology Collaboration (CKD-EPI) study equation.

Comorbidities were compared for categories of eGFR_cysC_/eGFR_crea_ ratio (> 0.7, 0.6–0.69 and < 0.6). Women with a ratio < 0.6 or between 0.6–0.69 were more likely to be on high blood pressure medications compared to those > 0.7 at age 75 and 80 (p = 0.043 and p = 0.015, respectively). However, no difference in distribution of cardiovascular disease or diabetes was observed at any time point (data not shown).

The extended follow-up allowed us to longitudinally investigate two consecutive five year time-windows. Longitudinally, just over half of those women (n = 80) with an eGFR_cysC_/eGFR_crea_ ratio < 0.6 at age 75 were alive and attended the five year follow-up (42/80) using the CKD-EPI study equation. Of these, the majority (n = 36) had ratios that had increased to 0.6 or higher. Correspondingly, at age 80, approximately one third (8/25) of women with eGFR_cysC_/eGFR_crea_ < 0.6 were alive and attended the next follow-up visit (age 85). Of these, 5 had an increased ratio of > 0.6. Results were similar for women with a ratio 0.6–0.69, were little less than half attending follow-up at age 80 had increased to a ratio > 0.7, and similarly from age 80–85 (data not shown).

**Figure 2:**
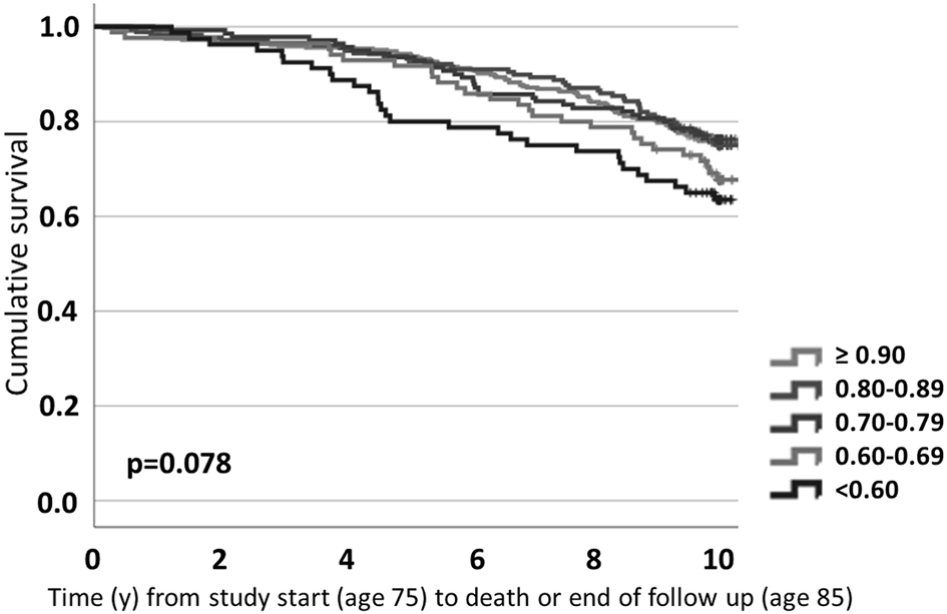
Ten year survival for five categories of eGFR_cysC_/eGFR_crea_ ratio. P-value calculated using the log Rank test.

The eGFR threshold for diagnosing chronic kidney disease (CKD) was based on the association between reduced function and mortality below a certain point (16). In this instance, to provide reference values for eGFR_cysC_/eGFR_crea_ ratio and evidence of a diagnostic threshold for SPS in averagely healthy older women, we investigated a range of ratios and the relationship to 10-year mortality. Using CKD-EPI study equation and data at age 75, the five categories were: eGFR_cysC_/eGFR_crea_ ratio ≥ 0.9 (n = 366), 0.8–0.89 (n = 178), 0.7–0.79 (n = 140), 0.6–0.69 (n = 85) and < 0.6 (n = 80)). Figure 2 shows survival between ages 75–85, a period in which a total of 221 women (26%) died. Although a difference in survival between the five categories was not observed (p = 0.078), women with a ratio < 0.6 had the lowest survival over the ten year period. Conversely, only those with an eGFR_cysC_/eGFR_crea_ ratio < 0.6 had increased mortality risk and this remained even after adjustment for covariates, including physical activity level (HR_adj_ 1.6, 95% CI [1.1–2.5]), Table 3). Taking eGFR_cysC_/eGFR_crea_ ratio as a continuous variable, mortality risk was reduced by half for every increase in ratio (HR_adj_ 0.5, 95% CI [0.2–0.9], p = 0.035).

**Table 3:** Association between eGFR_cysC_/eGFR_crea_ ratio at age 75 and 10-year mortality.

	eGFR_cysC_/eGFR_crea_ ratio (age 75)								
	≥ 0.9	0.8–0.89		0.7–0.79		0.6–0.69		< 0.6	
	*n* = 366	*n* = 178		*n* = 140		*n* = 85		*n* = 80	
		HR (95% CI)	p-value	HR (95% CI)	p-value	HR (95% CI)	p-value	HR (95% CI)	p-value
Unadjusted	1 (ref)	1.0 (0.7–1.5)	0.927	1.0 (0.7–1.5)	0.947	1.4 (0.9–2.1)	0.144	1.7 (1.1–2.6)	0.014
Model 1	1 (ref)	1.0 (0.7–1.4)	0.986	1.0 (0.6–1.4)	0.871	1.3 (0.8–2.0)	0.222	1.7 (1.1–2.6)	0.016
Model 2	1 (ref)	1.0 (0.7–1.5)	0.897	0.9 (0.6–1.4)	0.702	1.1 (0.7–1.8)	0.545	1.6 (1.1–2.5)	0.022

Model 1 adjusted for: diabetes, treatment for high blood pressure, cardiovascular disease and smoking.

Model 2 adjusted for: diabetes, treatment for high blood pressure, cardiovascular disease, smoking and self-reported physical activity (three categories: (1) bedbound or moving with the help of other people, (2) using walking aid, inside and out, (3) walk and exercise unhindered).

Using the CAPA/LM-rev study equations, at age 75, 3% had an eGFR_cysC_/eGFR_crea_ ratio < 0.6 and 7% a ratio < 0.7 (supplementary table S1), hence a lower SPS prevalence. However, the association to mortality was independent of study equation; using CAPA/LM-rev, women with a ratio < 0.6 had over two times increased mortality risk compared to women with a ratio > 0.9 (HR_adj_ 2.5, 95% CI [1.4–4.5], supplementary table S2). Survival over ten years based on the CAPA/LM-rev study equations is shown in supplementary figure S1.

## Discussion

We have investigated eGFR_cysC_/eGFR_crea_ ratio in this population based study of elderly women, and found that using the most common disease cutoff, one in ten women could be designated as having shrunken pore syndrome. The eGFR_cysC_/eGFR_crea_ ratio < 0.6 was associated with increased mortality risk over ten years.

In patient based cohorts the prevalence of SPS is reportedly between 0.2–11% depending on study equation (CKD-EPI, CAPA or LM-rev) and cutoff level^1,7,8,17^. In the 75 year old OPRA women, prevalence was 9–19% depending on cutoff. This high prevalence mirrors that in two heart disease populations^9,13^ and in patients referred for GFR examinations^6^. It is in stark contrast to a study by Purde et al. in healthy Swiss seniors aged 60 and over (mean age 72), which reported a considerably lower prevalence (< 1%), although study participants may have been healthier than the general senior population^4^. It should also be noted that a lower eGFR_cysC_/eGFR_crea_ ratio (CKD-EPI study equation) was reported in females than in males, a finding confirmed in a recent study linking SPS to female sex^11^. Given the considerably higher prevalence observed in the single sex, identically aged OPRA cohort it is clear that in terms of better understanding disruptions in the filtration process, age and comorbidities need to be taken into account. Since these two factors have a huge impact on kidney function in general, it is not surprising if this is also the case for diagnosing Shrunken pore syndrome, which may exist with both normal and reduced kidney function^6^. In addition, the GFR estimating equation is influential; the lower prevalence from CAPA/LM-rev in comparison to CKD-EPI study equations is in line with reports in patients with heart failure^13^ or undergoing heart surgery^7^.

At present, no generally accepted pathophysiological explanation for SPS exists, although shrinking of the pore size and thickening of the glomerular basement membrane (GBM) are the main hypotheses^1,5^. In diabetic glomerulosclerosis, thickening of the GBM may be reversible, at least in its early stages^18^ and it is possible that this is also the case in SPS, with data from pregnant women suggesting plasma levels of middle sized molecules returned to normal after delivery^3^. In the present study, there is a suggestion of reversal, since the majority of women with eGFR_cysC_/eGFR_crea_ ratio < 0.6 at age 75 had an increased ratio (> 0.6) at age 80. While caution is needed when interpreting the longitudinal data due to low numbers, it could be speculated that creatinine and cystatin C were affected by non-renal factors. Although the present study excluded women with sarcopenia, we cannot entirely rule out that any lessening of muscle mass with age may impact results. On the other hand, it may indicate the need for a more stringent definition of SPS, whereby only patients with a *persistent* ratio < 0.6 should be diagnosed. Such a diagnostic criteria is already employed for chronic kidney disease, requiring at least two consecutive measurements below cutoff, present for a given duration^19^.

This is the first study investigating the association between eGFR_cysC_/eGFR_crea_ ratio and mortality in a population based setting, and we have been able to demonstrate the long-term implications for mortality risk. In the OPRA cohort, women with an eGFR_cysC_/eGFR_crea_ ratio < 0.6 had a more than one and a half times higher risk of death at 10-years. This concurs with non-population based studies that found increased risk at cutoffs of both < 0.6 and < 0.7^6–8^, although follow-up times in these studies varies from one to just over five and a half years.

Interestingly, ratios between 0.6 and 0.69 were not associated with higher mortality, somewhat surprising in the light of a large study in patients referred for GFR determination; increased risk was already apparent at ratios of 1.0 to 0.85, with higher risk the lower the ratio^6^. It could be related to statistical power, however one explanation for these differences could be that the present study excluded women with sarcopenia. If sarcopenic women were included in analyses and categorized with a low eGFR_cysC_/eGFR_crea_ ratio, this could ‘falsely’ strengthen association with mortality since sarcopenia lowers plasma creatinine (therefore the probability of a low ratio), while sarcopenia itself is associated with mortality^20^. Another explanation for the difference in risk estimates could be the difference in patients (general health as well as age) referred for GFR determination compared to the population based OPRA cohort. Nevertheless, our results indicate that a ratio of < 0.6 may be most appropriate as a diagnostic threshold for SPS in elderly women. Our results also confirm suggestions that different clinical settings could require different cutoff levels^9,10^.

This is the first study investigating the association between eGFR_cysC_/eGFR_crea_ ratio and mortality risk in a population based setting, and limitations are acknowledged. Firstly, the OPRA cohort was originally designed to study fracture, therefore kidney related outcomes such as measured GFR and urine albumin were not assessed. Other studies report an increased mortality risk with SPS, even with normal GFR. This study does not have data on measured GFR, hence, it is for future studies to investigate this research question in a population based setting. While, as with measured GFR, data on urine albumin would have provided a more comprehensive picture of the participants kidney function, it is not essential for investigating prevalence and associated adverse outcomes of SPS. Urine albumin is important in assessing an individual’s kidney disease, not just to estimate kidney damage, but also for grading according to the 2012 Kidney Disease Improving Global Outcome guidelines. However, eGFR_cysC_/eGFR_crea_ ratio (SPS) is a newly described syndrome which does not take urine albumin into consideration. At the same time, the combination of SPS and urine albumin would probably provide a better prediction tool to identify individuals at risk of adverse outcomes. This will be addressed in future studies.

The longitudinal design of the OPRA cohort enables evaluation of kidney function over time and across multiple time points. Also, in contrast to other studies, OPRA includes information on sarcopenia and glucocorticoid treatment, two significant non-renal factors affecting creatinine and cystatin C and which allows us to establish an accurate picture of eGFR_cysC_/eGFR_crea_ ratio in the general elderly population. In addition, the long follow-up time of ten years makes this population based study unique. However, the present study lacks information on cause of death which would have been advantageous, considering eGFR_cysC_/eGFR_crea_ ratios of < 0.6 and < 0.7 have been associated with a variety of cardiovascular outcomes. This association cannot be investigated in the present setting, although the association between the eGFR_cysC_/eGFR_crea_ ratio and mortality was significant even after adjusting for a proxy for CVD (i.e. medications associated with cardiovascular diseases). Furthermore, the OPRA cohort does not have information on cognitive impairment, an important factor associated with both mortality and chronic kidney disease. However, given that all participants were community dwelling and participated in advance testing, the number of participants with severe cognitive impairment is probably low. While mortality and loss to follow-up due to reasons such as illness or moving to a senior home, lower the numbers at the follow-up visits, reduction in subject numbers is inevitable in this age group. Although, prevalence of SPS at follow up (age 80 and 85) should be interpreted with this in view, eGFR_cysC_/eGFR_crea_ ratio of < 0.6 is relatively stable over follow up.

Though participants in the OPRA cohort were randomly selected and no exclusion criteria applied, participants may be healthier than those who declined (21). This is not an not uncommon phenomenon in elderly populations (22), and it may indicate that the actual prevalence of eGFR_cysC_/eGFR_crea_ ratios < 0.6 and < 0.7 could be higher. Still, participation rate at age 75 was high (65%), increasing the likelihood we have a representative sample of average 75 year old women. Lastly, only Caucasian women of the same age were included in the OPRA cohort, which means that conclusions may not apply to men, other ages or other ethnicities. The authors look forward to future studies including both sexes in a diversified setting.

In conclusion, eGFR_cysC_/eGFR_crea_ ratio < 0.6 was common, affecting almost one in ten women at age 75. Mortality risk was increased at this threshold indicating it might be an appropriate cutoff in elderly women. Our findings prompt the need for further research, especially in other community based cohorts and including men.

## Material and methods

### Subjects

The Osteoporosis Prospective Risk Assessment (OPRA) cohort is a population based study originally designed as a fracture study^23^. From 1995 to 1999, 1604 75 year old women were randomly and without exclusion criteria selected from the city archives of Malmö and invited by letter to participate. Of these, 32 could not be reached despite several attempts, while 152 stated illness and 376 unwillingness as a cause for non-participation, leaving1044 women attending the baseline investigation (65% response rate). The first follow-up investigation took place after five years (age 80, n = 715) and the second after ten years (age 85, n = 382).

The present study uses data from those women with available cystatin C and creatinine values, corresponding to 963, 683 and 355 at respective visits. Missing values reflect a random loss across the cohort (for example, lack of serum, inability to provide a blood sample, hemolysis or failed analysis) and not a systematic error or selection. SPS should only be diagnosed in the absence of non-renal factors affecting cysC or creatinine. Hence, women with sarcopenia and/or taking glucocorticoids, or with missing values in any of these were excluded from the analyses, equating to 114 women at age 75 and 114 and 69 at age 80 and 85, respectively. This resulted in a final dataset of 849 women at baseline (age 75), with 569 and 286 at the five and ten year follow-ups, respectively. The study was approved by the regional ethical review board in Lund (Dnr: 2014804) and performed in accordance with the Helsinki declaration. All participants provided written informed consent.

### General chemistry

Blood samples were collected non-fasting before noon and stored at − 80°. Analyses were performed at the Department of Clinical Chemistry, Skåne University Hospital, Sweden. Details on analyses of cystatin C and creatinine has been described elsewhere^24,25^. In short, plasma cystatin C from all visits were analyzed in batch in 2015 using a Cobas auto-analyser adjusted to the international reference preparation ERM-DA 471/IFCC^26^ (Roche Diagnostics, Mannheim, Germany, CV ranging from 2.2 to 1.2%). Due to study duration and methodological updates, plasma creatinine was analyzed with a Beckman synchron LX20-4 (Beckman-Coulter, Ca, USA) or using the Cobas method. All creatinine samples have been adjusted to the Cobas method to ensure homogeneity and allow comparison between different time points and all values are IDMS traceable. Other biochemistry (plasma CRP, calcium, albumin, phosphate and serum PTH and 25OHD3) were analyzed in accordance with routine procedures at the time of study^27,28^.

### Kidney function and eGFR_cysC_/eGFR_crea_ ratio

Estimated Glomerular Filtration Rate (eGFR, mL/min/1.73 m^2^) was calculated using the Chronic Kidney Disease Epidemiology Collaboration (CKD-EPI) study equation based on cystatin C (eGFR_cysC_) and creatinine (eGFR_crea_)^29^, chosen because of its wide international use.

CKD-EPI study equation using creatinine:

- p-Cr ≤ 62 μmol/L: 144 × (p-Cr/(0.7 × 88.4))^−0.329^ × 0.993^Age^
- p-Cr > 62 μmol/L: 144 × (p-Cr/(0.7 × 88.4))^−1.209^ × 0.993^Age^

CKD-EPI study equation using cystatin C:

- p-CyC ≤ 0.8 mg/L: 133 × (p-cysC/0.8)^−0.499^ × 0.996^Age^ × 0.932
- p-CyC > 0.8 mg/L: 133 × (p-cysC/0.8)^−1.328^ × 0.996^Age^ × 0.932

For purpose of comparison to other studies, analyses based on eGFR calculated using the LM-rev and CAPA study equations^26,30^ were also performed (supplementary material). LM-rev and CAPA have been developed from individuals in the same geographical region. SPS is based on the ratio of eGFR_cysC_/eGFR_crea_, and while no disease definition yet exists, we used two cutoffs to estimate *prevalence* of the syndrome based on the available literature, eGFR_cysC_/eGFR_crea_ ratio < 0.6 or < 0.7. Since available data suggest risk estimation may differ based on the gradient of the eGFR_cysC_/eGFR_crea_ ratio, we also report prevalence and comorbidities for women with a ratio from 0.6 to 0.69.

### Sarcopenia

Sarcopenia, characterized by low muscle quantity and quality, was defined in accordance with the updated 2018 guidelines from the European Working Group on Sarcopenia in Older People^20^, as low muscle function *plus* low muscle mass. Muscle function was defined as low knee strength (< 175 Nms, equating to HGS < 16 kg). Muscle mass was measured using dual-energy x-ray absorptiometry. Low muscle mass was defined as < 5.5 kg/m^2^, through dividing appendicular skeletal lean mass (ASL), by height squared (ASL/height^2^, kg/m^2^). In the present study we excluded women with sarcopenia due to the risk of low muscle mass rendering a low plasma creatinine, and thus high eGFR_crea_, which could give a false low eGFR_cysC_/eGFR_crea_ ratio.

### Mortality

Date of death was obtained through the Swedish national population register and followed for ten years.

### Other assessments

At study start and each follow-up investigation, assessment included anthropometrics and a detailed questionnaire on medication, diseases and lifestyle. Information about previous CVD and high blood pressure (HBP) was not available at baseline and derived from medication information. HBP was defined as treatment with any anti-hypertensive and CVD as anti-hypertensive treatment in combination with an anticoagulant or lipid-modifying agent or treatment with only vasodilators (organic nitrates). Hence, CVD at baseline should be considered as an indirect measure of CVD. Information on CVD (i.e. myocardial infarction, angina pectoris or stroke) was available at the five and ten year follow-up. Data on physical activity were self-reported and categorized as: (1) bedbound or moving with help from other people, (2) using walking aid, inside and out and 3) walk and exercise unhindered.

### Statistics

Descriptive data is reported as mean with standard deviation (SD) or median with interquartile range (IQR). Differences in distribution of co-morbidities based on eGFR_cysC_/eGFR_crea_ ratio (three categories; > 0.7, 0.6–0.69 or < 0.6) were compared using Chi-squared. To explore eGFR_cysC_/eGFR_crea_ ratio over time, we investigated how many of the women with a ratio < 0.6 at age 75 maintained this ratio at age 80. We then repeated these calculations from age 80 to 85.

Ten year mortality risk was investigated for five categories of eGFR_cysC_/eGFR_crea_ ratio; > 0.9 (reference category), 0.8–0.89, 0.7–0.79, 0.6–0.69 and < 0.6 using cox proportional hazard models, unadjusted and adjusted for diabetes, high blood pressure, cardiovascular disease and current smoking status (model 1) and additionally for physical activity (model 2). The proportional hazards assumption was tested through log minus log plots. In addition, risk calculations with eGFR_cysC_/eGFR_crea_ ratio as a continuous variable were performed. Kaplan Meier curves were used to describe survival over ten years.

All statistical analyses were made using SPSS (IBM Corp. Released 2020. IBM SPSS Statistics for Windows, Version 27.0. Armonk, NY). A p-value below 0.05 was considered nominally significant.

### Statement of ethics

 Participants in the OPRA study gave written informed consent and study protocol has been approved by the Regional Ethical Review Board in Lund. The study was conducted in accordance with the Helsinki declaration.

## Supplementary Information

Supplementary Information.

## Data Availability

Data available on request from corresponding author Kristina Åkesson at e-mail: kristina.akesson@med.lu.se.
