# Supplementary material for: Impaired selective renal filtration captured by eGFRcysC/eGFRcrea ratio is associated with mortality in a population based cohort of older women
Source: Sci Rep. 2022 Jan 24;12:1273. doi: 10.1038/s41598-022-05320-w (PMC8786879; doi:10.1038/s41598-022-05320-w)
Supplement: Supplementary file 1 — Supplementary Information. [file 41598_2022_5320_MOESM1_ESM.docx]

Supplemental table 1. Prevalence of shrunken pore syndrome* at age 75 and the two follow up visits at age 80 and 85

|  | eGFR_cysC_/eGFR_crea_ ratio | |
| --- | --- | --- |
|  | *< 0.6* | *< 0.7* |
| *75* | 22 (3%) | 61 (7%) |
| *80* | 2 (0%) | 12 (2%) |
| *85* | 2 (1%) | 8 (3%) |

*SPS defined using the CAPA and LM-rev study equations

Age 75; n=849, age 80 n=569, age 85; n=286

Supplemental table 2. Association between the eGFR_cysC_/eGFR_crea_ ratio* at age 75 and mortality followed for ten years (from age 75-85)

|  | eGFR_cysC_/eGFR_crea_ ratio (age 75) | | | | | | | | |
| --- | --- | --- | --- | --- | --- | --- | --- | --- | --- |
|  | ≥0.9 | 0.8-0.89 | | 0.7-0.79 | | 0.6-0.69 | | <0.6 | |
|  |  | HR (95% CI) | p-value | HR (95% CI) | p-value | HR (95% CI) | p-value | HR (95% CI) | p-value |
|  | (n=633) | (n=99) |  | (n=56) |  | (n=39) |  | (n=22) |  |
| Unadjusted | 1 (ref) | 1.1 (0.7-1.6) | 0.806 | 1.7 (1.1-2.7) | 0.029 | 1.2 (0.7-2.3) | 0.497 | 2.9 (1.6-5.1) | <0.001 |
| Model 1 | 1 (ref) | 1.0 (0.7-1.6) | 0.895 | 1.6 (1.0-2.6) | 0.043 | 1.3 (0.7-2.4) | 0.407 | 2.6 (1.4-4.7) | 0.002 |
| Model 2 | 1 (ref) | 1.0 (0.7-1.6) | 0.896 | 1.4 (0.9-2.3) | 0.144 | 1.3 (0.7-2.3) | 0.457 | 2.5 (1.4-4.5) | 0.003 |

* CAPA and LM-rev study equations were used to define the eGFR_cysC_/eGFR_crea_ ratio

Model 1 adjusted for: diabetes, treatment for high blood pressure, cardiovascular disease and smoking (yes/no).

Model 2 adjusted for: diabetes, treatment for high blood pressure, cardiovascular disease, smoking (yes/no) and physical activity level.

**Supplemental figure 1.** Ten year survival for five categories of the eGFR_cysC_/eGFR_crea_ ratio


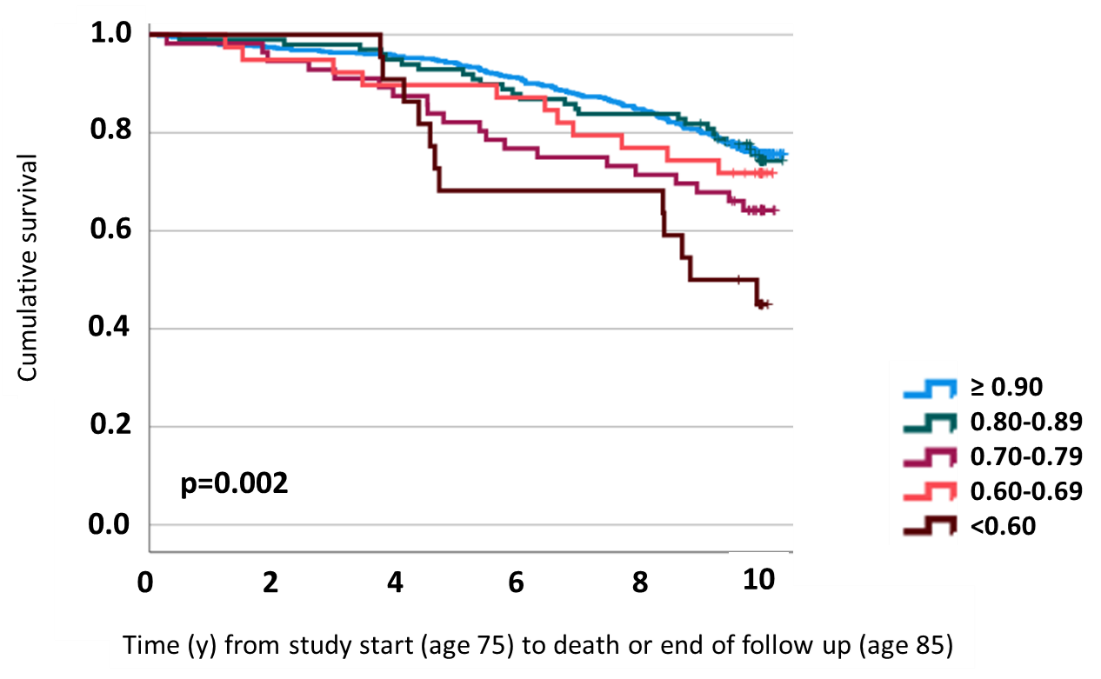


P-value calculated using the log Rank test. CAPA and LM-rev study equations were used to define the eGFR_cysC_/eGFR_crea_ ratio
